# Transcriptome Dynamics and Cell Dialogs Between Oocytes and Granulosa Cells in Mouse Follicle Development

**DOI:** 10.1093/gpbjnl/qzad001

**Published:** 2023-12-06

**Authors:** Wenju Liu, Chuan Chen, Yawei Gao, Xinyu Cui, Yuhan Zhang, Liang Gu, Yuanlin He, Jing Li, Shaorong Gao, Rui Gao, Cizhong Jiang

**Affiliations:** Key Laboratory of Spine and Spinal Cord Injury Repair and Regeneration of Ministry of Education, Orthopaedic Department of Tongji Hospital, School of Life Sciences and Technology, Tongji University, Shanghai 200092, China; Clinical and Translational Research Center of Shanghai First Maternity and Infant Hospital, Shanghai Key Laboratory of Signaling and Disease Research, School of Life Sciences and Technology, Tongji University, Shanghai 200092, China; Frontier Science Center for Stem Cell Research, Tongji University, Shanghai 200092, China; Clinical and Translational Research Center of Shanghai First Maternity and Infant Hospital, Shanghai Key Laboratory of Signaling and Disease Research, School of Life Sciences and Technology, Tongji University, Shanghai 200092, China; Frontier Science Center for Stem Cell Research, Tongji University, Shanghai 200092, China; Clinical and Translational Research Center of Shanghai First Maternity and Infant Hospital, Shanghai Key Laboratory of Signaling and Disease Research, School of Life Sciences and Technology, Tongji University, Shanghai 200092, China; Frontier Science Center for Stem Cell Research, Tongji University, Shanghai 200092, China; Key Laboratory of Spine and Spinal Cord Injury Repair and Regeneration of Ministry of Education, Orthopaedic Department of Tongji Hospital, School of Life Sciences and Technology, Tongji University, Shanghai 200092, China; Key Laboratory of Spine and Spinal Cord Injury Repair and Regeneration of Ministry of Education, Orthopaedic Department of Tongji Hospital, School of Life Sciences and Technology, Tongji University, Shanghai 200092, China; Key Laboratory of Spine and Spinal Cord Injury Repair and Regeneration of Ministry of Education, Orthopaedic Department of Tongji Hospital, School of Life Sciences and Technology, Tongji University, Shanghai 200092, China; Department of Epidemiology and Biostatistics, Jiangsu Key Lab of Cancer Biomarkers, Prevention and Treatment, Collaborative Innovation Center for Cancer Personalized Medicine, School of Public Health, Nanjing Medical University, Nanjing 210029, China; Department of Epidemiology and Biostatistics, Jiangsu Key Lab of Cancer Biomarkers, Prevention and Treatment, Collaborative Innovation Center for Cancer Personalized Medicine, School of Public Health, Nanjing Medical University, Nanjing 210029, China; Clinical and Translational Research Center of Shanghai First Maternity and Infant Hospital, Shanghai Key Laboratory of Signaling and Disease Research, School of Life Sciences and Technology, Tongji University, Shanghai 200092, China; Frontier Science Center for Stem Cell Research, Tongji University, Shanghai 200092, China; Clinical and Translational Research Center of Shanghai First Maternity and Infant Hospital, Shanghai Key Laboratory of Signaling and Disease Research, School of Life Sciences and Technology, Tongji University, Shanghai 200092, China; Frontier Science Center for Stem Cell Research, Tongji University, Shanghai 200092, China; Key Laboratory of Spine and Spinal Cord Injury Repair and Regeneration of Ministry of Education, Orthopaedic Department of Tongji Hospital, School of Life Sciences and Technology, Tongji University, Shanghai 200092, China; Frontier Science Center for Stem Cell Research, Tongji University, Shanghai 200092, China

**Keywords:** Folliculogenesis, Oocyte, Granulosa cell, Transcriptome, Cell dialog

## Abstract

The development and maturation of follicles is a sophisticated and multistage process. The dynamic gene expression of oocytes and their surrounding somatic cells and the dialogs between these cells are critical to this process. In this study, we accurately classified the oocyte and follicle development into nine stages and profiled the gene expression of mouse oocytes and their surrounding granulosa cells and cumulus cells. The clustering of the transcriptomes showed the trajectories of two distinct development courses of oocytes and their surrounding somatic cells. Gene expression changes precipitously increased at Type 4 stage and drastically dropped afterward within both oocytes and granulosa cells. Moreover, the number of differentially expressed genes between oocytes and granulosa cells dramatically increased at Type 4 stage, most of which persistently passed on to the later stages. Strikingly, cell communications within and between oocytes and granulosa cells became active from Type 4 stage onward. Cell dialogs connected oocytes and granulosa cells in both unidirectional and bidirectional manners. TGFB2/3, TGFBR2/3, INHBA/B, and ACVR1/1B/2B of TGF-β signaling pathway functioned in the follicle development. NOTCH signaling pathway regulated the development of granulosa cells. Additionally, many maternally DNA methylation- or H3K27me3-imprinted genes remained active in granulosa cells but silent in oocytes during oogenesis. Collectively, Type 4 stage is the key turning point when significant transcription changes diverge the fate of oocytes and granulosa cells, and the cell dialogs become active to assure follicle development. These findings shed new insights on the transcriptome dynamics and cell dialogs facilitating the development and maturation of oocytes and follicles.

## Introduction

Oocytes originate from primordial germ cells and develop within follicles. Oocyte and follicle development is a multistage process. There are various methods to classify the development stages. The distinguishing criteria include the volume of the follicles and the number of granulosa layers surrounding the oocyte. Terms of primordial, primary, secondary follicles, and so on have been used to describe the stages of follicle development. However, these methods are not accurate enough to define the stages of oocyte and follicle development. Therefore, the accurate transcriptome dynamics in the course of the oocyte and follicle development is lacking.

Follicle development (folliculogenesis) is a well-coordinated process including oocyte growth (oogenesis) and maturation, as well as the proliferation of granulosa cells. Oocytes and their surrounding granulosa cells do not develop in an isolated mode. A previous study has hypothesized that oocytes interact with granulosa cells during oogenesis [1]. The crosstalk between oocytes and granulosa cells operates via two types of mechanisms, *i.e.*, signal transduction mediated by (1) cytokines or growth factors and (2) gap junctions which permit small molecules exchanging [2]. The bidirectional communications between oocytes and granulosa cells are vital for oocyte maturation and follicle growth [3]. Although progresses have been made in our understanding of oocyte–somatic cell dialogs [2,4], it is still poorly understood what factors are involved in this event and how the communication is regulated.

The genomes of sperm and oocytes have distinct epigenetic landscapes. After fertilization, their epigenetic landscapes are remodeled and largely become the same except for some regions with unequal epigenetic modifications, that is, genome imprinting. Both DNA methylation and histone modifications [trimethylation of histone H3 at lysine 9 (H3K9me3) and lysine 27 (H3K27me3)] can cause imprinting [5,6]. Imprinting plays a critical role in embryo development. The genomes of somatic cells and gametes are packaged with different epigenetic landscapes. Epigenetic remodeling after fertilization leads to abnormal imprinting in somatic cell nuclear transfer (SCNT) embryo genome compared with the naturally fertilized embryo genome. This is a key factor for low SCNT efficiency [5,7,8]. It has been reported that maternal chromatins inherit various histone modifications from oocytes [9,10]. However, the expression profiles of imprinted genes during oogenesis remain elusive.

In this study, we accurately classified the stages of oocyte and follicle development and collected oocytes and their surrounding granulosa cells and cumulus cells at each stage. An ultra-low-input RNA sequencing (RNA-seq) method was applied to determine their transcriptomic profiles. We revealed the close communications between oocytes and granulosa cells, as well as the distinct expression patterns of imprinted genes in oocytes and their surrounding somatic cells during oogenesis. These findings greatly improve our understanding of the transcriptome dynamics and molecular dialogs regulating oocyte and follicle development.

## Results

### Global transcriptome profiling of mouse oocytes and granulosa cells

**Figure 1:**
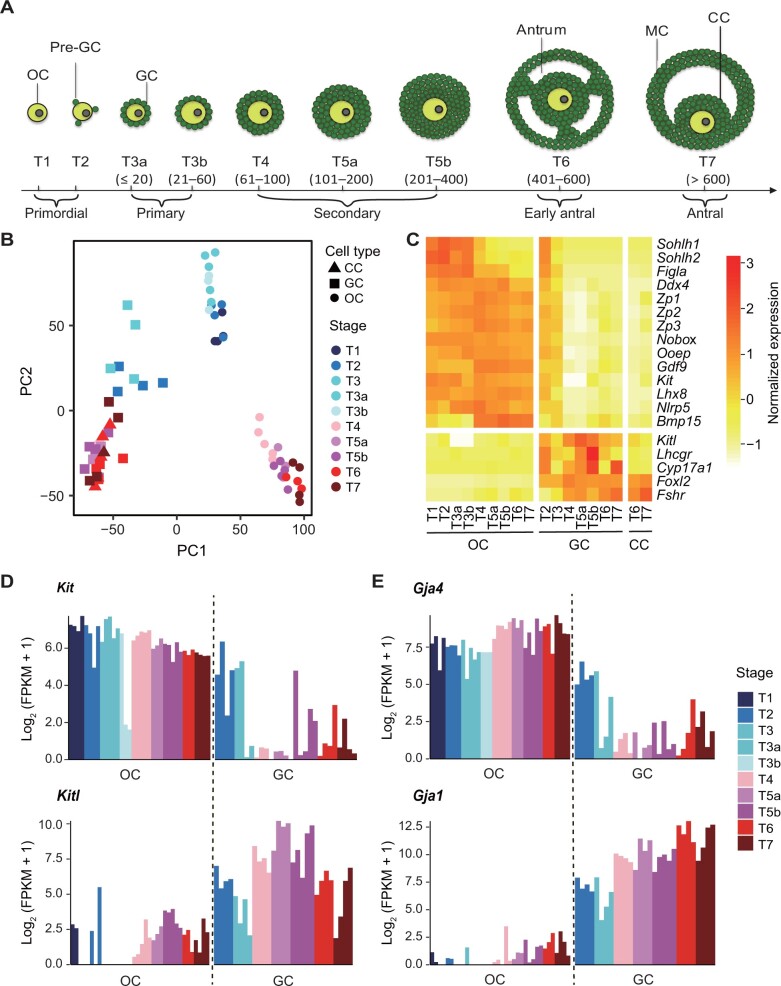
The global transcriptome profiling of mouse OCs and GCs **A**. Illustration of follicle development stages at which OCs, GCs, and CCs are collected. **B**. PCA of the transcriptome data from OCs, GCs, and CCs. **C**. Heatmap showing the normalized expression levels of OC, GC, and CC marker genes. **D**. and **E**. The normalized expression levels of representative genes involved in the two types of OC–GC/CC crosstalks: the interactions between ligands and receptors (*Kitl* and *Kit*) (D) and the molecular exchange through gap junctions (*Gja1* and *Gja4*) (E). OC, oocyte; Pre-GC, pre-granulosa cell; GC, granulosa cell; CC, cumulus cell; MC, mural cell; PC, principal component; PCA, principal component analysis; FPKM, fragments per kilobase of exon model per million mapped fragments; T, type.

To investigate the development of oocytes and the crosstalk between oocytes and their neighboring granulosa cells, we obtained mouse oocytes, granulosa cells, and cumulus cells at sequential stages of oocyte and follicle development (**Figure 1**A), which was accurately defined based on the size of the oocyte and the follicle and the morphology of the follicle [11]. Then, RNA-seq of oocytes, granulosa cells, and cumulus cells was performed using an ultra-low-input approach, respectively. The replicates were highly reproducible (Figure S1A). The unsupervised hierarchical clustering of the transcriptomic profiles showed that granulosa and cumulus cells at different stages were clustered together and so were oocytes (Figure S1B). Interestingly, the oocyte cluster was comprised of two sub-clusters: early stages (Type 1 to Type 3b) and other stages (Type 4 to Type 7). Similarly, granulosa and cumulus cells of Type 4 to Type 7 were clustered together (Figure S1B). Consistently, the principal component analysis (PCA) of the transcriptomic profiles clearly separated oocytes and granulosa cells (Figure 1B). Pseudotime analysis of gene expression reflected the development trajectory of oocytes and granulosa cells (Figure S1C). These findings indicate that oocytes and granulosa cells have different expression profiles and their expression programs are highly dynamic in the course of oocyte and follicle development. Additionally, in line with the fact that cumulus cells are derived from granulosa cells, they have similar expression profiles.

Transcription silence and maternal messenger RNA (mRNA) accumulation take place in oogenesis. Moreover, the balance between transcription and degradation is broken. Therefore, it is important to add spike-in RNAs in the study of oocyte transcriptomes. We repeated RNA-seq by adding spike-in RNAs for Type 4- and Type 7-stage oocytes. The comparisons showed high correlations between the transcriptomes of oocytes with and without spike-in RNA addition (Figure S1D). The expression patterns of the marker genes for oocytes, granulosa cells, and cumulus cells calculated by the RNA-seq data with and without spike-in RNAs were highly similar (Figure 1C, Figure S1E). Together, the transcriptomes of oocytes in our study are reliable in measuring mRNA levels.

Next, we examined the cell type-specific expression patterns of the known markers for oocytes and granulosa cells [12–14]. The results showed that oocyte maker genes were specifically highly expressed in oocytes, so did granulosa cell maker genes (Figure 1C). Notably, most of the oocyte marker genes were also mildly or highly expressed in granulosa cells at Type 2 stage, and the expression levels decreased at Type 3 stage. This is likely that there are only a few cells (pre-granulosa cells) attached to the oocyte surface at Type 2 stage. The pre-granulosa cells may share similar expression profiles of some genes with oocytes. PCA of the maker genes for oocytes (*Ddx4* and *Ooep*) [15,16] and granulosa cells (*Foxl2* and *Cyp19a1*) [17,18] confirmed their cell type-specific expression patterns during follicle development (Figure S2A and B). We also identified several genes whose expression levels in the course of follicle development were similar to those of the marker genes for oocytes and granulosa cells, respectively (Figure S2C and D). Immunofluorescence staining confirmed their cell type-specific expression (Figure S2E). These genes could be the candidate marker genes for oocytes and granulosa cells.

There exist many dialogs between oocytes and their surrounding somatic cells, through which molecular exchange takes place between oocytes and granulosa cells/cumulus cells [2]. For example, the KIT ligand (*Kitl*) gene was specifically expressed in granulosa cells/cumulus cells, and its protein product acted on oocyte KIT receptors [19,20]. In line with this, *Kit* was highly expressed in oocytes across all stages and very lowly expressed in granulosa cells, while the expression pattern of *Kitl* was opposite (Figure 1D). Similarly, *Gja4* encodes connexin-37 in oocytes responsible for oocyte–granulosa cell gap junctions, while *Gja1* encodes exonnexin-43 responsible for the gap junctions between granulosa cells [21,22]. *Gja4* was highly expressed in oocytes across all stages and very lowly expressed in granulosa cells, while the expression pattern of *Gja1* was opposite (Figure 1E).

Collectively, our transcriptomic data recapitulated the course of oocyte and follicle development, the expression patterns of the marker genes for oocytes and granulosa cells, and the gene pairs responsible for the dialogs between oocytes and granulosa cells. Therefore, the transcriptomic data are reliable and appropriate to interrogate the development of oocytes and the crosstalk between oocytes and their surrounding granulosa cells/cumulus cells.

### Type 4 is an important turning stage during oocyte and follicle development

**Figure 2:**
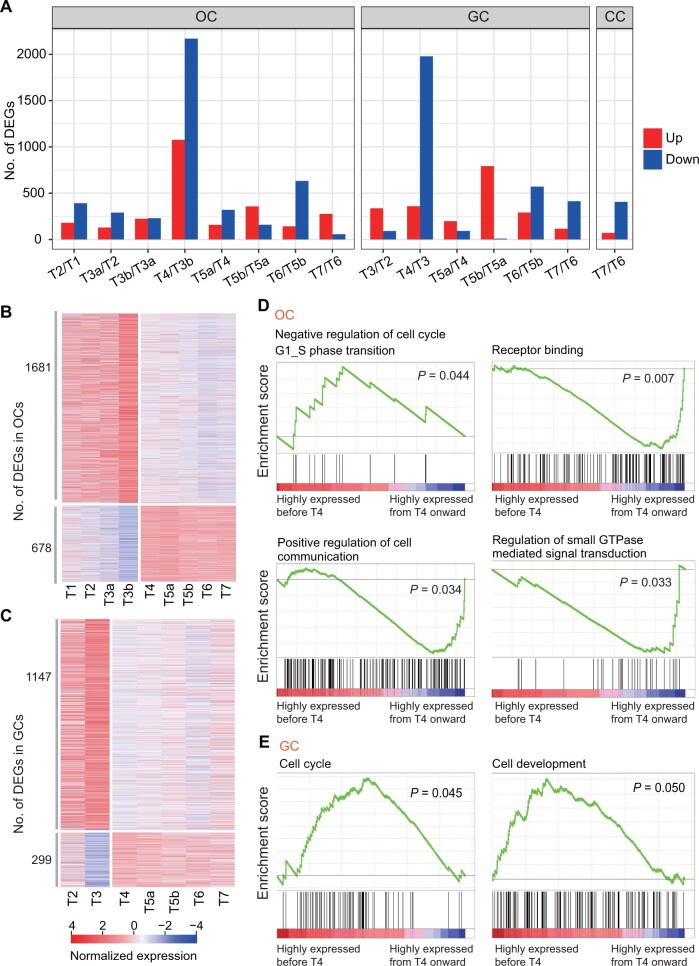
Gene expression dynamics of OCs and GCs **A**. Bar plots showing the number of DEGs between consecutive follicular development stages in OCs, GCs, and CCs, respectively. **B**. Heatmap showing the expression levels of the DEGs specific between T4 and T3b OCs in (A) across follicle development stages. **C**. Heatmap showing the expression levels of the DEGs specific between T4 and T3 GCs in (A) across follicle development stages. **D**. GSEA results showing the functions of the 1681 (the top-left plot) and the 678 (the rest of three plots) T4-specific DEGs continuously highly expressed in OCs before T4 or from T4 onward in (B). **E**. GSEA results showing the functions of 1147 T4-specific DEGs continuously highly expressed in GCs before T4 in (C). DEG, differentially expressed gene; GSEA, Gene Set Enrichment Analysis.

To explore gene expression changes during oocyte and follicle development, we identified the differentially expressed genes (DEGs) between two adjacent stages for oocytes, granulosa cells, and cumulus cells [fold change (FC) ≥ 2 or ≤ 0.5; *P* < 0.05], respectively. Interestingly, both oocytes and granulosa cells had the largest number of DEGs between Type 4 and Type 3 stages (**Figure 2**A). This finding suggests that there occur the largest changes in gene expression from Type 3 to Type 4, indicating that Type 4 is a critical stage in oocyte and follicle development. Additionally, very few of the DEGs are a persistent DEG at each stage compared with the previous stage (Figure S3A and B). This indicates that most DEGs are stage specific.

In order to understand the functions of the DEGs at Type 4 stage, we retained the DEGs specifically at Type 4 stage by removing the genes that were also DEGs at other stages. There were a total of 2359 DEGs in oocytes and 1446 DEGs in granulosa cells specifically at Type 4 stage. Surprisingly, these DEGs remained highly expressed either before Type 4 or from Type 4 onward (Figure 2B and C). Gene Set Enrichment Analysis (GSEA) of the 1681 Type 4-specific DEGs continuously highly expressed in oocytes before Type 4 revealed functional enrichment in negative regulation of cell cycle, while the 678 Type 4-specific DEGs continuously highly expressed from Type 4 onward were enriched in positive regulation of cell communication, receptor binding, and regulation of small GTPase mediated signal transduction (Figure 2D). For granulosa cells, GSEA of the 1147 Type 4-specific DEGs continuously highly expressed in granulosa cells before Type 4 revealed enrichment in cell cycle and cell development (Figure 2E). These results suggest that cell communications between oocytes and granulosa cells become active from Type 4 onward.

### Gene expression dynamics of oocytes and granulosa cells during oogenesis

**Figure 3:**
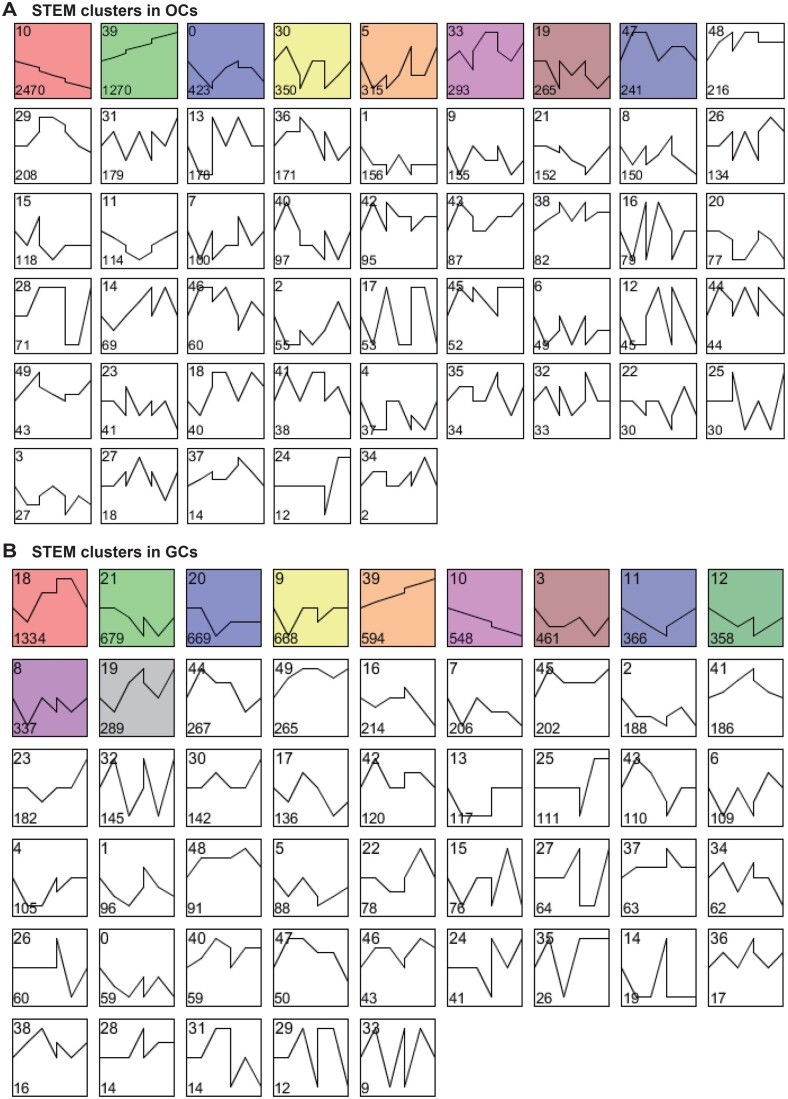
Clustering of DEGs based on their expression levels in OCs and GCs STEM clustering of all DEGs in the development of OCs (**A**) and GCs (**B**). The colored clusters showed statistically significant expression profiles (*P* ≤ 0.05, multiple hypothesis test). The number in the top-left corner indicates the cluster number. The number in the bottom-left corner indicates gene count assigned to a cluster. The clusters are ordered by gene count. STEM, Short Time-series Expression Miner.

To obtain knowledge about the gene expression dynamics during oocyte and follicle development, we applied the Short Time-series Expression Miner (STEM) to identify expression patterns [23]. STEM clustering analysis of the gene expression profiles of oocytes generated 50 clusters, only 8 of which were statistically significant (*P* ≤ 0.05) (**Figure 3**A). Only 3 statistically significant clusters (Clusters 10, 39, and 0) showed a regular expression pattern. Gene expression persistently decreased in Cluster 10, while the expression of genes in Cluster 39 was continuously up-regulated (Figure 3A). In contrast, gene expression levels first decreased, next increased, and then decreased again in Cluster 0. The rest of clusters showed a more complex pattern with pervasive fluctuation (Figure 3A). Together, only 10.27% of expressed genes were persistently up-regulated during oocyte development, while 19.97% were continuously down-regulated. The rest of genes had a fluctuating expression profile (Figure S3C). STEM analysis of the gene expression profiles of granulosa cells also generated 50 clusters, 11 of which were statistically significant (*P* < 0.05). Similarly, most clusters had an irregular expression pattern (Figure 3B). Interestingly, the percentage of genes with continuously increasing or decreasing expression in granulosa cells was much lower than that in oocytes, while the percentage of genes with irregular expression patterns was much higher than that in oocytes (Figure S3C and D). This implies that gene expression is more dynamic in granulosa cells than in oocytes during oocyte and follicle development.

We further performed Gene Ontology (GO) analysis of the statistically significant clusters. The GO analysis of the three statistically significant clusters of oocyte genes identified enrichment for meiosis-related GO terms: sister chromatin segregation, meiotic cell cycle process, nuclear division, female meiotic division, and so on (Figure S3E). The similar GO analysis also revealed that the statistically significant clusters of granulosa cell genes were enriched for sister chromatid segregation, ovulation, oogenesis, mitotic cell cycle process, female gamete generation, oocyte maturation, nuclear division, and so on (Figure S3F). These findings clearly show that gene transcription in both oocytes and granulosa cells is dynamically programmed to facilitate oocyte development.

### Gene expression difference between oocytes and granulosa cells

**Figure 4:**
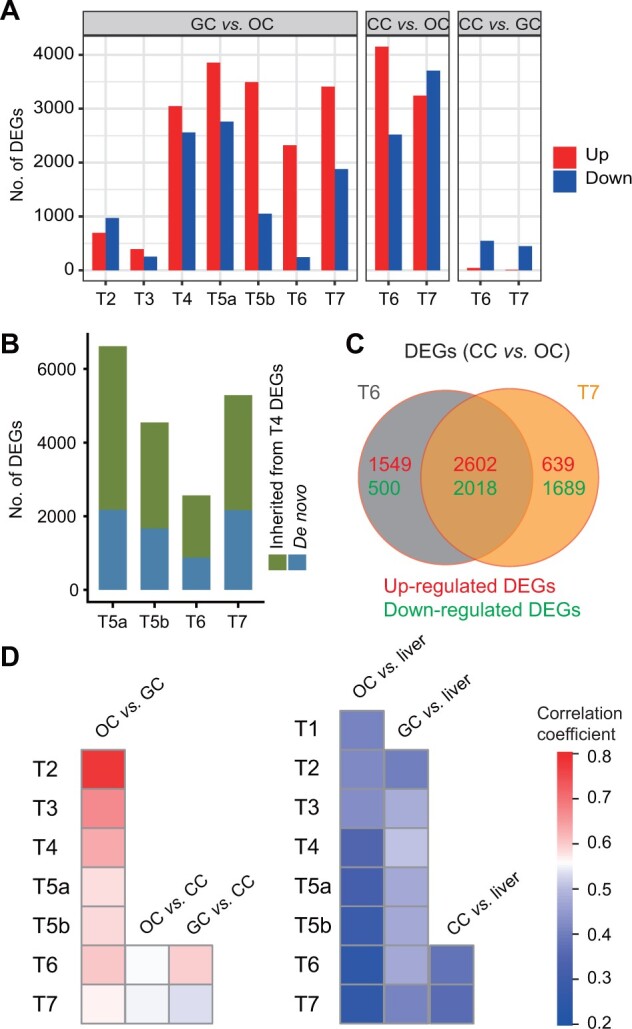
Comparison of gene expression between OCs and different types of cells **A**. Bar plots showing the number of DEGs between GCs and OCs, CCs and OCs, and CCs and GCs across developmental stages. **B**. Number of the DEGs between GCs and OCs at later stages inherited from the DEGs at T4 stage. **C**. Venn diagram showing overlap of the DEGs between CCs and OCs at T6 and T7 stages. **D**. The pairwise correlations of gene expression between OCs, GCs, CCs, and liver cells.

We next pairwise compared gene expression between oocytes and surrounding somatic cells. The results showed that the number of DEGs between oocytes and granulosa cells precipitously increased from Type 4 onward with a little fluctuation (**Figure 4**A). There also existed a large number of DEGs between oocytes and cumulus cells at Type 6 and Type 7 stages. Contrarily, there were only some DEGs between granulosa cells and cumulus cells (Figure 4A). Interestingly, more than half of DEGs at each stage of Type 5a to Type 7 were inherited from the DEGs of Type 4 in the granulosa cell *vs.* oocyte comparison (Figure 4B). Of note, there existed a certain number of *de novo* DEGs at each stage (Figure 4B, Figure S4A). This suggests that Type 4 stage is a critical time point when sufficient difference in gene expression may gradually diverge oocytes and granulosa cells in terms of development and molecular functions. Similarly, more than half of DEGs between oocytes and cumulus cells at Type 6 and Type 7 stages were overlapped, while both stages had many unique DEGs (Figure 4C). These results together suggest that gene transcription programs of the surrounding somatic cells gradually deviate from those of oocytes by partially inheriting the divergence of the previous stage and establishing new difference at the same time.

Further analysis showed that the transcription profiles of oocytes, granulosa cells, and cumulus cells were pairwise highly correlated. The correlation decreased along oocyte and follicle development. In contrast, their gene expression profiles were very lowly correlated with that of liver (Figure 4D, Figure S4B). This further suggests that oocytes and their surrounding granulosa cells and cumulus cells have relatively similar gene expression programs.

### Dialogs between oocytes and granulosa cells along follicle development

**Figure 5:**
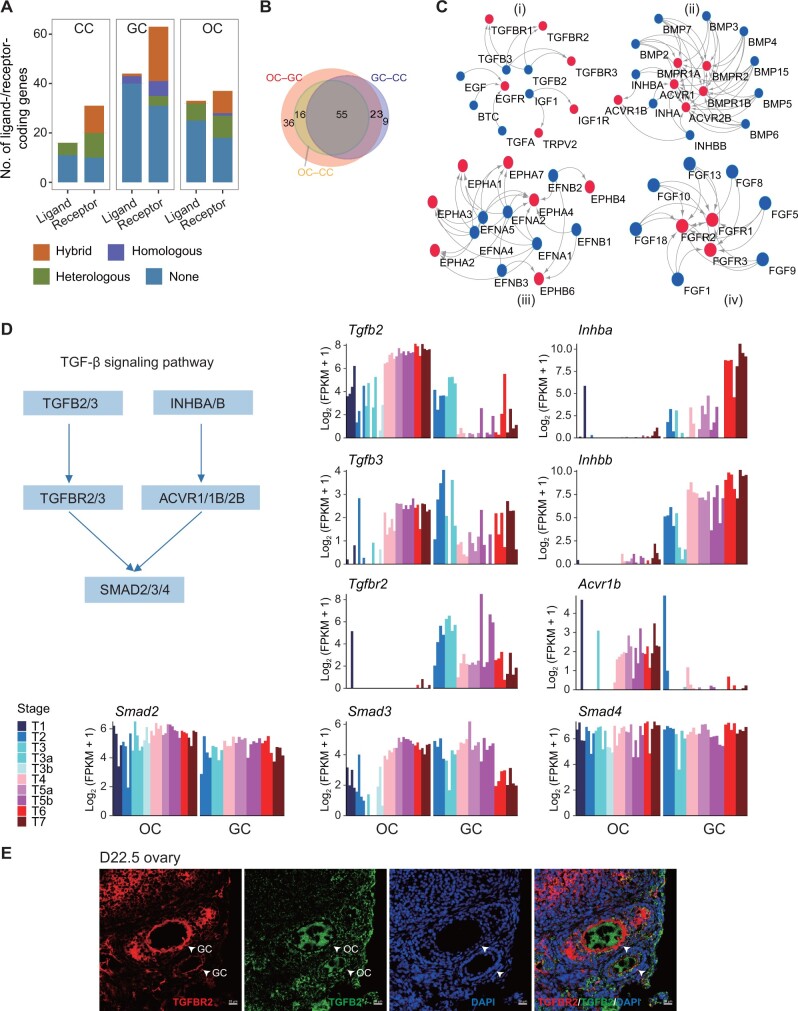
The cell crosstalks between OCs and GCs during follicle development **A**. Bar plot showing the number of categorized ligand- and receptor-coding genes that are expressed in OCs, GCs, and CCs, respectively. Homologous indicates that ligand–receptor pairs only form within the given cell type. Heterologous indicates that ligand–receptor pairs only form between the given cell type and at least one of the other two cell types. Hybrid indicates that ligand–receptor pairs belong to both “Homologous” and “Heterologous” groups. None indicates that no ligand–receptor pairs form, *i.e.*, only ligand- or receptor-coding gene is expressed but not both. **B**. Venn diagram showing the overlap of all ligand–receptor pairs that form between any two cell types. **C**. Networks showing ligand–receptor pairs enriched between any two cell types, indicating potential cell–cell dialogs in oogenesis. Nodes indicate ligands (blue) or receptors (red), and edges indicate ligand–receptor pairs with the arrows go from ligands to receptors. **D**. The TGF-β signaling pathway involved in OC–GC crosstalk. The bar plots showing the expression levels of genes encoding the components of the TGF-β signaling pathway in OCs and GCs across follicle development stages. **E**. Immunofluorescence staining of TGFB2–TGFBR2 ligand–receptor pair in D22.5 mouse ovary. The ligand TGFB2 is expressed in the OCs while the receptor TGFBR2 is expressed in GCs. Scale bar, 20 μm. D22.5, 22.5 days after birth; DAPI, 4′6-diamidino-2-phenylindole.

The dialogs between oocytes and their surrounding somatic cells are pivotal to folliculogenesis. To comprehensively explore the crosstalk between oocytes and granulosa cells, we collected a non-redundant set of 336 known interacting ligand–receptor pairs from previous studies (see Materials and methods for details) consisting of 124 ligands and 113 receptors. A ligand or receptor was considered to be expressed in oocytes if its coding gene showed a maximal expression level [fragments per kilobase of exon model per million mapped fragments (FPKM)] ≥ 5 at least at one stage. Same rule was used for definition of ligand or receptor expression in granulosa cells and cumulus cells. A dialog existed if the coding genes of a ligand–receptor pair were expressed in oocytes and/or surrounding somatic cells. Most known ligand–receptor pairs didn’t exist in oocytes and/or surrounding somatic cells. Very few expressed pairs were homologous, *i.e*., both ligand- and receptor-coding genes were expressed in the same type of cells. A certain number of expressed pairs were heterologous, *i.e.*, ligand- and receptor-coding genes were expressed in the different type of cells. Most expressed pairs were hybrid, *i.e*., both homologous and heterologous (**Figure 5**A). There existed 139 ligand–receptor pairs consisting of 54 ligands and 44 receptors (Figure 5B, Figure S5A and B). Most dialogs pairwise between oocytes, granulosa cells, and cumulus cells were shared in common. Interestingly, ligand–receptor pairs between oocytes and cumulus cells were a subset of the dialogs between oocytes and granulosa cells (Figure 5B). There were only 9 unique ligand–receptor pairs between granulosa cells and cumulus cells (Figure 5B, Figure S6A). Of note, ∼ 50% of the dialogs in our study were confirmed in either of the databases CellChat [24] or CellCall [25]. Moreover, our method, CellChat, and CellCall all identified many pathways important to follicle development such as TGF-β, NOTCH, and ACTIVIN signaling pathways (Figure S6B). These results suggest that there exist extensive dialogs between oocytes and their surrounding somatic cells, which play an important role in folliculogenesis.

In order to reveal whether there exists interplay between these ligand–receptor pairs, we put the 139 ligand–receptor pairs together and used Cytoscape [26] to draw interaction networks among them. The results showed that most dialogs formed networks belonging to several protein families, including TGFB, BMP, FGF, EFNA/B, and interleukin (receptor) (Figure 5C, Figure S6C). For example, it was reported that oocytes promoted cumulus cells to express EGFR which in turn enabled cumulus cells to respond to EGF-like peptides (amphiregulin, epiregulin, and betacellulin) induced by luteinizing hormone (LH) in granulosa cells. Moreover, the oocyte-expressed GDF9 and BMP15 also controlled the expression of EGFR [2,27]. This indicates that dialogs exist within and between protein families, which together facilitate maturation of follicles.

### Signaling pathways involved in oocyte–granulosa cell crosstalk during folliculogenesis

Many pathways drive follicle maturation and activation [3]. To understand the interplays between oocytes and granulosa cells, we analyzed the gene expression of ligands, receptors, and targets that were the components of key signaling pathways. We examined the gene expression of the key components of the NOTCH signaling pathway. The ligand-coding genes *Dll3*, *Jag1*, and *Jag2* were highly expressed in oocytes across the developmental stages, while the receptor-coding genes *Notch2* and *Notch3* were highly expressed throughout the development of granulosa cells. The downstream target genes *Dtx3*, *Dtx3l*, and *Hes1* were also highly expressed in granulosa cells across the developmental stages (Figure S7A and B). This indicates that oocytes mediate the development of granulosa cells through NOTCH signaling pathway. This finding is consistent with a previous study [3].

Previous studies have shown that the TGF-β signaling pathway plays a critical role in controlling folliculogenesis through different members (GDF9 and BMP15), which have different roles in the follicle development and function at different stages [3,28]. Therefore, we also examined the gene expression of the important members of the TGF-β signaling pathway during folliculogenesis. The ligand-coding genes *Tgfb2* and *Tgfb3* of the TGF-β signaling pathway were highly expressed throughout oocyte development, while the receptor-coding gene *Tgfbr2* was highly expressed throughout granulosa cell development (Figure 5D, Figure S7B). Immunofluorescence staining confirmed the cell type-specific expression pattern of TGFB2–TGFBR2 ligand–receptor pair (Figure 5E, Figure S7C). In contrast, the ligand-coding genes *Inhba* and *Inhbb* were highly expressed across the development of granulosa cells, whereas the receptor-coding gene *Acvr1b* was highly expressed in oocytes from Type 4 (*i.e*., secondary follicle) onward. The target genes of the SMAD superfamily were highly expressed throughout the maturation of both oocytes and granulosa cells (Figure 5D, Figure S7B). It has been reported that SMAD signaling sustains proliferation and differentiation of granulosa cells [2]. Deletion of *Smad2* and/or *Smad3* in granulosa cells greatly decreased female fertility [29]. These results together suggest that the TGF-β signaling pathway regulates follicle maturation through bidirectional ligand–receptor interactions between oocytes and granulosa cells. Some interactions were also stage specific. Of note, TGFB2/3, TGFBR2/3, INHBA/B, and ACVR1/1B/2B are new members of the TGF-β signaling pathway functioning in the development of follicles. A previous study reported that mutations in *TGFBR3* were highly correlated in human ovarian failure, suggesting that *TGFBR3* could be used as a susceptibility marker gene for ovarian failure aetiology [30].

### Expression dynamics of maternal-effect genes and maternally imprinted genes during oocyte development

**Figure 6:**
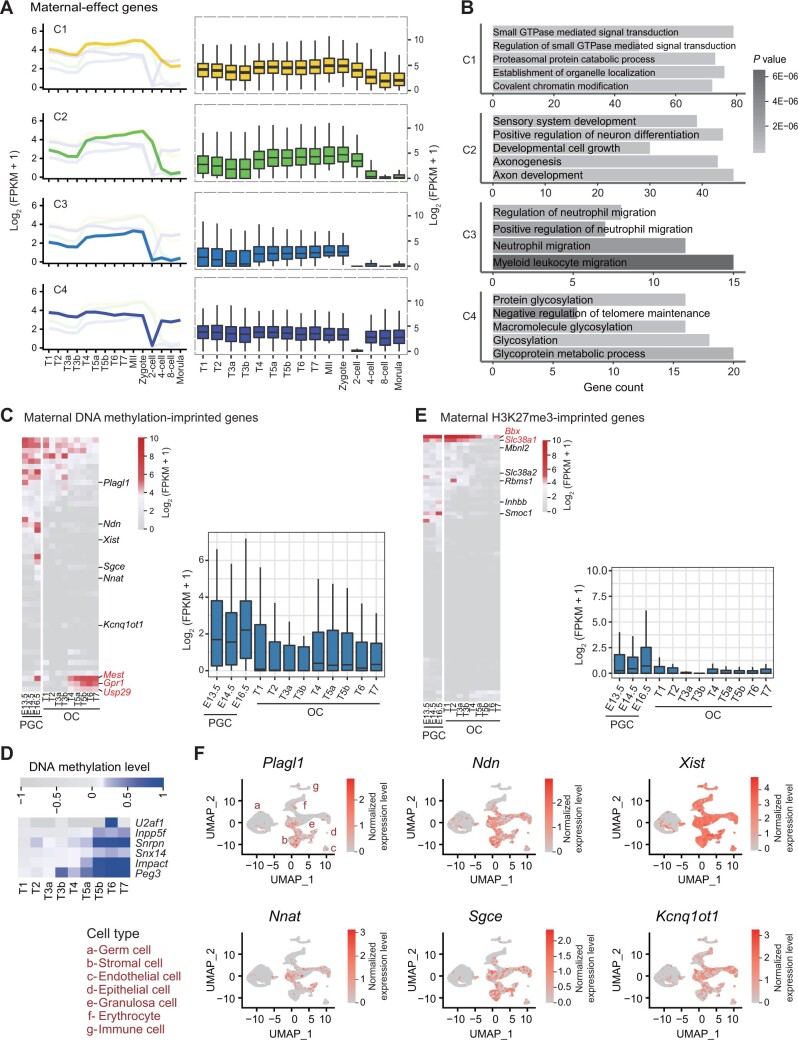
Expression profiles of maternal-effect genes and maternally imprinted genes along the development of PGCs, OCs, and early embryos **A**. Both line plots (left) and box plots (right) showing the expression levels of the maternal-effect genes during oogenesis and early embryo development. The maternal-effect genes are clustered into four groups (C1–C4) by the similarity of expression patterns. **B**. GO enrichment analysis of each group of genes in (A). **C**. Both heatmap and box plot showing the expression levels of selected DNA methylation-dependent maternally imprinted genes in PGCs and OCs. **D**. DNA methylation dynamics in the promoters of the imprinted genes that are gradually silenced during OC development. The DNA methylation data are from GSE111684. **E**. Both heatmap and box plot showing the expression levels of selected H3K27me3-dependent maternally imprinted genes in PGCs and OCs. **F**. Expression of the selected maternally DNA methylation-imprinted genes (indicated in C) in different ovarian cell types. The single-cell RNA-seq data are from GSE134339. The full imprinted gene lists are given in Table S1. GO, Gene Ontology; PGC, primordial germ cell; RNA-seq, RNA sequencing; UMAP, Uniform Manifold Approximation and Projection.

The maternal-to-zygotic transition (MZT) is a critical developmental event during which zygotic gene products start to take over developmental control from mRNAs and proteins provided by the oocyte. To characterize the expression patterns of maternal-effect genes during oocyte development, we downloaded RNA-seq data of mouse preimplantation embryos that had been generated in previous studies [31]. We identified 3753 maternal-effect genes whose expression levels (FPKM) were > 2 in MII oocytes and at least two times higher than that at one or more later stages (zygote, 2-cell embryo, 4-cell embryo, 8-cell embryo, or morula). These maternal-effect genes were classified into four groups (C1–C4) by unsupervised clustering analysis (Figure S8A). Their expression levels were dramatically reduced from 2- or 4-cell stage. This was in agreement with that many maternally provided mRNAs were degraded in mouse 2-cell embryos where the major wave of zygotic genome activation started [32,33]. Then, we examined the expression patterns of the four groups of maternal-effect genes. The results showed that all the maternal-effect genes had an intermediate expression level early in Type 1 oocytes. Their expression levels gradually increased from Type 4 stage and reached the maximum in MII oocytes (**Figure 6**A). The GO annotation of these genes revealed that the C1 set of genes were enriched for small GTPase mediated signal transduction, proteasomal protein catabolic process, establishment of organelle localization, *etc*. The C2 set of genes were enriched for sensory system development, positive regulation of neuron differentiation, axonogenesis, axon development, *etc*. The C3 set of genes were enriched for regulation of neutrophil migration, positive regulation of neutrophil migration, neutrophil migration, *etc*. The C4 set of genes were enriched for protein glycosylation, macromolecule glycosylation, glycosylation, glycoprotein metabolic process, *etc*. (Figure 6B). This indicates that the maternal-effect genes have diverse functions.

Imprinting genes play important roles in embryo development. The maternally imprinted genes are the genes whose maternal alleles are silenced in zygotes. To understand when the maternally imprinted genes are silenced during oocyte development, we collected 50 maternally imprinted genes from the imprinted gene database (Geneimprint). These genes were imprinted by high DNA methylation. Most of these genes were silenced in the female primordial germ cells (PGCs) and remained silenced during oocyte development (Figure 6C). Of note, *Mest*, *Gpr1*, and *Usp29* were mildly or highly expressed at Type 4 and later stages. This implies that they may not be *bona fide* maternally imprinted genes. Consistent with the gradually silenced imprinted genes, the DNA methylation levels in the promoters gradually increased during oocyte development (Figure 6D). In contrast, ∼ 50% of the maternally imprinted genes were expressed in granulosa and cumulus cells during oocyte development (Figure S8B). This suggests that most maternally imprinted genes are silenced not after fertilization but at the beginning of oocyte development.

Recently, a new type of maternally imprinted genes independent of DNA methylation were identified. The maternal alleles were imprinted by H3K27me3 and also played a critical role in embryo development [5]. What is the scenario for the expression of these maternally H3K27me3-imprinted genes during oocyte development? The results showed that all these genes except for *Bbx* and *Slc38a1* were silenced in the female PGCs and remained silenced during oocyte development (Figure 6E). Notably, most the maternally H3K27me3-imprinted genes were also silenced in granulosa and cumulus cells during oocyte development (Figure S8C). This suggests that the maternally H3K27me3-imprinted genes are also silenced not after fertilization but at the beginning of oocyte development. Further analysis showed that both maternally DNA methylation- and H3K27me3-imprinted genes were expressed in other somatic cell types in ovary using ovary single-cell transcriptome data (Figure 6F, Figure S8D). The different expression patterns of these two types of maternally imprinted genes in granulosa and cumulus cells during oocyte development imply the potential subtle difference of their functions. Further studies are required to address this issue.

To examine the interspecies complexity between human and mouse, we collected 1440 human maternal-effect genes [3]. There were only 517 homologous maternal-effect genes between human and mouse (Figure S9A). However, their expression patterns during oocyte development were different between human and mouse (Figure S9B). Comparison of the maternally DNA methylation-imprinted genes revealed less than half of them common in mouse and human. Most common maternally imprinted genes are silenced during both mouse and human follicle development (Figure S9C and D). These findings imply that there exist sufficient variations in transcription program during follicle development between human and mouse.

## Discussion

A previous study has reported that less than 1% of the follicles develop to maturity and successfully ovulate [34]. Successful oogenesis and oocyte maturation depend on close cooperation and crosstalk between the oocyte and its surrounding granulosa cells and cumulus cells. Here, we comprehensively explored the communications between oocytes and granulosa cells based on receptor–ligand pairs and identified novel factors and pathways regulating follicle development. For example, TGFB2/TGFB3–TGFBR2 and INHBA/INHBB–ACVR1B co-targeted SMAD superfamily genes to communicate oocytes and granulosa cells through a bidirectional interaction. We also identified several protein families such as TGFB, BMP, FGF, and EFNA/B that were also involved in the regulation of follicular development. These new candidate factors and signaling pathways could provide valuable clues for future functional studies related to folliculogenesis and may provide new therapeutic strategies for ovarian abnormalities and treatment of infertility.

Maternal-effect genes are critical to early embryo development. Interestingly, our results showed that maternal-effect genes already had prominent transcription levels at T1 stage. Maternal-effect genes showed different expression patterns after great degradation by 2-cell stage. Basically, there were two groups of maternal-effect transcripts: stable and unstable. The unstable maternal-effect transcripts both in mice and *Drosophila* were reported to be enriched for cell cycle-related GO terms [35,36]. In contrast, the stable ones in *Drosophila* were highly enriched for GO terms related to RNA transaction [36]. Consistently, pre-loaded RNA and protein synthesis is required for the rapid fly early embryo development. However, the GO terms related to RNA transaction were enriched in zygotic transcripts not in maternal-effect transcripts in mice [35]. This is consistent with the more slowly development of mouse embryos. Our study also showed that there were a large number of different maternal-effect transcripts between human and mouse. Together, maternal-effect genes function in early embryo development in a species-specific manner.

Correct genomic imprinting is essential to embryo development. Our results showed that many maternally imprinted genes (marked by either DNA methylation or H3K27me3) were expressed in granulosa cells and cumulus cells while they were silenced in oocytes. This suggests different imprinting between granulosa/cumulus cells and oocytes, which raises critical epigenetic barriers resulting in low development rate of SCNT embryos. The differentially maternally imprinted genes in granulosa/cumulus cells may be candidates to improve SCNT efficiency through epigenetic editing.

## Conclusion

Our study provides the first comprehensive characterization of the transcription program and the communications between oocytes and surrounding granulosa cells/cumulus cells during mouse folliculogenesis. Gene expression changes precipitously increase at Type 4 stage when the fate of oocytes and granulosa cells/cumulus cells diverges. Cell dialogs also become active at Type 4 stage and assure follicle development. The different expression patterns of maternally imprinted genes between oocytes and granulosa cells are established at the beginning of folliculogenesis. These findings improve our understanding of the processes of mammalian follicle development and female germ cell maturation, and shed new lights on the therapeutic strategies targeting follicular abnormalities and infertility.

## Materials and methods

### Isolation and collection of mouse oocytes, granulosa cells, and cumulus cells

According to size of oocyte, total cell number, and morphology of the follicles, the growing follicles were classified into different types: primordial follicles (Type 1 and Type 2), primary follicles (Type 3), secondary follicles (Type 4 and Type 5), early antral follicles (Type 6), and late antral follicles (Type 7) [11]. In this study, oocytes, granulosa cells, and cumulus cells were obtained from B6D2F1 (C57BL/6 × DBA2) mice that were at embryonic day 18.5 (E18.5) and postnatal days 2.5, 6.5, 12.5, 17.5, and 22.5 (P2.5, P6.5, P12.5, P17.5, and P22.5), respectively. Details of sample collection for subsequent sequencing analysis were listed as below.

For primordial follicles, the Type 1 and Type 2 oocytes and Type 2 granulosa cells were obtained by digesting the ovaries from E18.5 and P2.5 mice using 0.25% trypsin (Catalog No. 15-050-065, Gibco, Carlsbad, CA) and 2 mM ethylenediaminetetracetic acid (EDTA; Catalog No. 03690-100ML, Sigma, St. Louis, MO) supplemented with 0.1% DNase I, and kept at 37°C. Then, the tissues were repeatedly drawn in and out of a Pasteur pipet to dissociate the cells into a single cell suspension. Type 1 naked oocytes were isolated from E18.5 germ cell cyst. In Type 2 follicles which were obtained from P2.5 mice, oocytes were just surrounded by several cuboidal granulosa cells.

For primary follicles, the Type 3a and Type 3b oocytes and Type 3 granulosa cells were obtained by digesting the P6.5 mouse ovaries with crude collagenase and DNase I in Dulbecco’s Phosphate-Buffered Saline (DPBS; Catalog No. C14190500CP, Life technologies, Waltham, MA). At Type 3a and Type 3b stages, only a single layer of cuboidal granulosa cells surrounded oocytes, and Type 3b oocytes were relatively larger in diameter than Type 3a oocytes.

For secondary follicles, the Type 4, Type 5a, and Type 5b oocytes and granulosa cells were mainly obtained from P12.5 mice. Two layers of granulosa cells surrounded the Type 4 oocytes, while six or seven layers of granulosa cells surrounded the Type 5b oocytes. Type 5a follicles exhibited an intermediate morphology between the Type 4 and Type 5b follicles.

For antral follicles, we collected the Type 6 and Type 7 oocytes, granulosa cells, and cumulus cells from P17.5 and P22.5 mice, respectively, as follows: using syringe needles to puncture the antral follicles; then using finely drawn glass pipets to strip the cumulus cells from the oocytes, and repeatedly drawing the cells into the fine pipets; washing the cumulus cell-denuded oocytes in EmbryoMax CZB medium (Catalog No. MR-019-D, Millipore, Hayward, CA) to remove somatic cells with care. The Type 6 antral follicles firstly generated a small fluid-filled antrum. The Type 7 oocytes were tightly surrounded by the cumulus cells. The mural granulosa cells lined the inner wall of the antral follicles.

### Immunofluorescence staining

Mouse ovarian tissues at 11.5 and 22.5 days after birth (D11.5 and D22.5) were fixed in 4% paraformaldehyde (PFA) for 2 h or 4–5 h at 4°C depending on the stage. After fixation, the ovaries were transferred to 30% sucrose for cryoprotection, and then embedded and frozen in Tissue-Tek OCT (Catalog No. 4583, Sakura Finetek, Tokyo, Japan) for cryosectioning. Fixed ovarian tissues were prepared as 8-μm cryosections, and then immunofluorescence staining was performed as previously described [37]. Briefly, all samples were incubated with primary antibodies [TGF-βII (Catalog No. MAB73461-SP, R&D Systems, Minneapolis, MN); TGF-βRII (Catalog No. sc-17799, Santa Cruz, Dallas, TX); ZNF541 (Catalog No. sc-515333, Santa Cruz); RAB34 (Catalog No. sc-376710, Santa Cruz)] overnight at 4°C. Sections were washed, incubated with Alexa Fluor 488 (AF488; Catalog No. A-11006, Thermo Fisher Scientific, Waltham, MA)/Alexa Fluor 594 (AF594; Catalog No. A-21207, Thermo Fisher Scientific) conjugated secondary antibodies for 45 min at room temperature, and mounted in prolong anti-fade reagent with 4′,6-diamidino-2-phenylindole (DAPI; Catalog No. C1002, Beyotime, Shanghai, China). Confocal imaging was performed with Zeiss LSM 880 confocal microscopes (Carl Zeiss AG, Oberkochen, Germany) and analyzed with Zeiss ZEN blue edition.

### Construction of RNA-seq libraries

The collected oocytes, granulosa cells, and cumulus cells were transferred into lysis buffer by a mouth pipette, respectively. The cytoplasmic lysate was directly used for reverse transcription. A poly(A) tail was added to the 3′ end of the first-strand complementary DNA (cDNA) by terminal deoxynucleotidyl transferase (TdT), and 18–20 cycle amplifications of the total cDNA were performed. Then, we used Covaris sonicator (Catalog No. S220, Covaris, Woburn, MA) to fragment the amplified cDNA. Next, the KAPA HyperPrep Kit (Catalog No. KK8504, Roche, Basel, Switzerland) was used to generate RNA-seq libraries following the manufacturer’s instructions. Lastly, sequencing was performed with paired-end 100-bp or 125-bp protocol on a HiSeq 2500 or 2000 (Illumina, San Diego, CA) at Berry Genomics Corporation.

As for construction of RNA-seq libraries of Type 4 and Type 7 oocytes with spike-in RNAs, the diluted ERCC RNA Spike-In Mix (Catalog No. 4456740, Thermo Fisher Scientific) was added to lysis buffer as spike-in RNAs. The rest of steps were the same as the above construction of RNA-seq library without adding spike-in RNAs.

### Processing and analysis of RNA-seq data

Firstly, the adaptor sequences, low-quality bases, and reads shorter than 50 bp were removed by Cutadapt (v1.18) [38]. Then, we mapped the trimmed clean reads to mouse reference genome (mm10) by HISAT2 (v2.1.0) [39] with parameters “--dta-cufflinks --no-discordant”. Next, we quantified gene expression as FPKM by StringTie (v1.3.4d) [40]. Genes with FPKM < 1 in all samples were filtered, and FPKM values of replicates were averaged.

PCA and hierarchical clustering analysis were performed in R studio (https://www.rstudio.com/), and ggplot2 was used to draw graphs.

### Comparison of RNA-seq data with and without spike-in RNAs

After mapping described as above, the reads with spike-in RNAs were counted using featureCounts (v2.0.0). After that, gene counts were normalized using ERCC spike-in reads as controls using “RUVg” method from R package RUVSeq (v1.20.0). And we transformed count values into FPKM values for subsequent analysis. Based on the normalized expression, the Pearson correlations between the transcriptomes with and without ERCC spike-in RNAs were calculated.

### Identification of DEGs

We characterized the DEGs between two adjacent stages during oogenesis and between two different types of cells at the same stage by Cufflinks (v1.3.0) [41]. The significant DEGs were selected with *P* < 0.05 and expression FC ≥ 2 or ≤ 0.5.

### Analysis of time-series gene expression patterns

STEM [23] was used to explore gene expression patterns on time-series for oocytes and granulosa cells with default parameters. Then, STEM results were classified into four categories as non-significant (*P* > 0.05), significantly continuously up (*P* ≤ 0.05), significantly continuously down (*P* ≤ 0.05), significantly fluctuating (*P* ≤ 0.05).

### Functional annotation analysis

To identify the enriched functions of a given gene set, we performed GO enrichment analyses in the Database for Annotation, Visualization and Integrated Discovery (DAVID) (v6.8) [42] and GSEA (v3.0) [43].

### Prediction and visualization of potential oocyte–granulosa/cumulus cell crosstalk

Firstly, we downloaded known ligand–receptor pairs from Database of Ligand–Receptor Partners (DLRP; http://dip.doe-mbi.ucla.edu/dip/DLRP.cgi) and IUPHAR (http://www.guidetopharmacology.org/) databases. We obtained 366 previously published ligand–receptor pairs in total. Secondly, we required that the expression of ligand- and receptor-coding genes should be ≥ 5 FPKM at least in one of oocytes, granulosa cells, and cumulus cells. A ligand–receptor pair is active only if both ligand- and receptor-coding genes are expressed. Thirdly, we built cell–cell communication matrix and generated the network graph of ligand–receptor pairs by Cytoscape (v3.7.0) [26] to display overall oocyte–granulosa/cumulus cell crosstalk.

### Ligand–receptor interaction analysis

CellChat (v1.6.0) R package [24] was used to calculate the cell–cell interactions of known ligand–receptor pairs between oocytes and granulosa cells. Firstly, “createCellChat” function was used to make CellChat object, and CellChatDB.mouse was loaded. Then, “computeCommunProb” function was used to compute the communication probability. Next, “computeCommunProb-Pathway” function was used to infer cell–cell communications of different signaling pathways with the parameter “*P* value <= 0.05”. Finally, “plotGeneExpression” function and Venn (v1.11) R package were used to visualize these results.

CellCall (v0.0.0.9) R package [25] was also used to infer the cell–cell communications between oocytes and granulosa cells. Firstly, count matrix of oocytes and granulosa cells was inputted into “CreateNichConObject” function to create the CellCall object. Then, the cell–cell communication score of a ligand–receptor pair was calculated by “TransCommuProfile” function with default parameters.

### Analysis and graphic display of DNA methylation data

DNA methylation [reduced representation bisulfite sequencing (RRBS)] data were downloaded from Gene Expression Omnibus (GEO: GSE111684). DNA methylation level in the promoter (±1 kb to transcription start site) was counted as the mean of DNA methylation levels at the CpGs within the promoter.

### Analysis and graphic display of single-cell RNA-seq data

We downloaded the Seurat object from GEO (GEO: GSE134339) [44,45] and converted it to SingleCellExperiment object by “as.SingleCellExperiment” function. Then, “Feature-Plot” function from Seurat (v1.0.5) R package was used to visualized marker expression.

### Trajectory analysis

Trajectory analysis of follicle development was performed using the Monocle (v2.18.0) R package [46]. Firstly, “new_cell_data_set” function was used to create the new cell_data_set object based on the count matrix. Then, “preprocess_cds” function was used for normalization and initial dimensional reduction with parameters: norm_method = “log”, method = “PCA”. After the dimensional reduction by Uniform Manifold Approximation and Projection (UMAP), “learn_graph” and “order_cells” functions were used to predict the trajectory. Finally, “plot_cells” function was used to plot the cells along with their trajectories.

### Identification of potential maternal-effect genes

To identify candidate maternal-effect genes, we downloaded RNA-seq data at six time points (MII, zygote, 2-cell embryo, 4-cell embryo, 8-cell embryo, and morula) during the development of mouse embryos. First, we set MII as a reference and calculated the FC of gene expression between other samples and MII. The maternal-effect genes should meet the following two requirements: (1) the expression level (FPKM) in MII is larger than 2; and (2) the expression level in at least one of the stages (zygote, 2-cell embryo, 4-cell embryo, 8-cell embryo, and morula) is less than half of that in MII. We identified 3753 maternal-effect genes.

## Ethical statement

All experiments were approved by the Biological Research Ethics Committee of Tongji University (Approval No. TJAB03221114) and conducted in full compliance with the University of Health Guide for the Care and Use of Laboratory Animals.

## Supplementary Material

qzad001_Supplementary_Data

## Data Availability

The RNA-seq data of oocytes, granulosa cells, and cumulus cells generated in this study have been deposited in the Genome Sequence Archive [47] at the National Genomics Data Center, Beijing Institute of Genomics, Chinese Academy of Sciences China / National Center for Bioinformation (GSA: CRA001613), and are publicly accessible at https://ngdc.cncb.ac.cn/gsa.

## CRediT author statement

**Wenju Liu:** Conceptualization, Formal analysis, Visualization, Writing – original draft. **Chuan Chen:** Investigation. **Yawei Gao:** Investigation, Supervision, Conceptualization. **Xinyu Cui:** Data curation. **Yuhan Zhang:** Data curation. **Liang Gu:** Project administration. **Yuanlin He:** Resources. **Jing Li:** Resources. **Shaorong Gao:** Supervision. **Rui Gao:** Investigation, Visualization, Writing – original draft. **Cizhong Jiang:** Supervision, Conceptualization, Visualization, Writing – review & editing. All authors have read and approved the final manuscript.
