# Supplementary material for: Transcriptome Dynamics and Cell Dialogs Between Oocytes and Granulosa Cells in Mouse Follicle Development
Source: Genomics Proteomics Bioinformatics. 2023 Dec 6;22(2):qzad001. doi: 10.1093/gpbjnl/qzad001 (PMC11423849; doi:10.1093/gpbjnl/qzad001)
Supplement: qzad001_Supplementary_Data [file qzad001_supplementary_data.zip › Supplementary material captions.docx]

**Supplementary material**

**Figure S1 Correlations of RNA-seq replicates and clustering of global gene expressions**

**A.** Pearson correlations of RNA-seq replicates for OCs, GCs, and CCs. **B.** Hierarchical clustering of gene expression profiles of all samples. **C.** Trajectories of OCs and GCs/CCs constructed by Monocle. **D.** Pearson correlations of the transcriptomes with and without spike-in RNAs. **E.** Heatmap showing the normalized expression levels of OC, GC and CC marker genes. T4_S and T7_S use RNA-seq with spike-in RNA. The rest use RNA-seq without spike-in RNAs.

**Figure S2 Expression patterns of individual marker genes**

**A****.** and **B**. PCA plots showing expression patterns of OC (A) and GC (B) marker genes. **C****.** and **D.** PCA plots showing expression of candidate marker genes of OCs (C) and GCs (D). **E.** Immunofluorescence staining of the candidate marker genes for GCs and OCs in the D22.5 mouse ovary (postnatal 22.5 days). The circled region indicates GCs (top) and the OC (bottom), respectively. Scale bar: 50 μm.

**Figure S3 Dynamics and functional annotations of DEGs in OCs and GCs along follicle development**

**A.** and **B**. Alluvial plots showing the expression dynamics of all DEGs defined in Figure 2A in the development of OCs (A) and GCs (B). **C.** Pie plot summarizing the portion of the genes in the STEM clusters defined in Figure 3A: persistent increasing expression (cluster 39), persistent decreasing expression (cluster 10), other significant clusters with fluctuating expression (clusters 0, 5, 19, 30, 33, and 47), all other non-significant clusters during OC development. **D.** Pie plot summarizing the portion of the genes in the STEM clusters defined in Figure 3B: persistent increasing expression (cluster 39), persistent decreasing expression (cluster 10), other significant clusters with significant fluctuating expression (clusters 3, 8, 9, 11, 12, 18, 19, 20, and 21), all other non-significant clusters with non-significant change during GC development. **E.** and **F.** Significantly enriched GO terms (biological processes) for selected gene sets defined in STEM clustering (Figure 3A and B) of expression profiles in OCs (E) and GCs (F). The STEM cluster number is given in the grey box.

**Figure S4 Comparison of gene expressions and functional annotations of DEGs**

**A.** Venn diagram showing overlap of the up-regulated(left) or down-regulated (right) DEGs between GCs and OCs at T4, T5a, T5b, T6, and T7 stages. **B.** Top panel: the scatter plots showing the pairwise correlation of gene expressions between OCs, GCs, and CCs at T7 stage. Bottom panel: the scatter plots showing the correlation of gene expressions between liver cells and OCs/GCs/CCs at T7 stage.

**Figure S5 Expression profiles of ligand**–**receptor pairs in OCs and GCs/CCs**

**A.** and **B.** Heatmaps showing the expression levels of the ligand–receptor pair genes. (A) ligands are expressed in OCs; (B) receptors are expressed in OCs.

**Figure S6 Overlap of cell dialogs identified by different methods**

**A.** Heatmap showing the expression levels of the genes encoding GC–CC specific ligand–receptor pairs. **B.** Venn diagram showing the overlap of the dialogs between OCs and GCs/CCs identified in our study, CellChat, and CellCall. **C.** Two newly identified networks for which ligand–receptor pairs are enriched, indicating potential cell dialogs in oogenesis. Nodes: ligands (blue) or receptors (red); edges: the arrow goes from ligands to receptors.

**Figure S7 Potential cell dialogs and signaling pathway involved in crosstalk between OCs and GCs**

**A.** The NOTCH signaling pathway involved in OC–GC crosstalk. The bar plots showing the expression levels of the component genes of the NOTCH signaling pathway in OCs and GCs across follicle development stages. **B.** The expression levels of the componential genes in the representative signaling pathways (TGF-β, ACTIVIN, and NOTCH) in OCs and GCs/CCs. Expression data is from CellChat. **C.** Immunofluorescence staining of TGFB2–TGFBR2 ligand–receptor pair in D11.5 mouse ovary. The ligand TGFB2 is expressed in the OCs while the receptor TGFBR2 is expressed in GCs. Scale bar: 20 μm.

**Figure S8 Expression profile of maternal-effect genes and maternally imprinted genes along PGCs, OC, and early embryo development**

**A.** Heatmap showing the fold change of maternal-effect gene expression in early embryo development, with MII as reference. **B.** Both Heatmap and box plots showing the expression levels of selected DNA methylation-dependent maternally imprinted genes in PGCs, GCs/CCs, and early embryos. **C.** Both Heatmap and box plots showing the expression levels of selected H3K27me3-dependent maternally imprinted genes in PGCs, GCs/CCs, and early embryos. **D.** Expression of the selected maternally H3K27me3-imprinted genes (indicated in C) in different ovarian cell types. The single-cell RNA-seq data is from GSE134339. The full imprinted gene lists are given in the Table S1.

**Figure S9 Comparison of maternal-effect genes and maternally imprinted genes along OC embryo development between human and mouse**

**A.** Venn diagram showing overlap of the maternal-effect genes between human and mouse. **B.** Heatmap showing the expression patterns of the common maternal-effect genes in mouse and human in the OC development. **C.** Venn diagram showing overlap of the maternally DNA methylation-imprinted genes between human and mouse. **D.** Heatmap showing the expression patterns of the common maternally DNA methylation-imprinted genes in mouse and human in the OC development.

**Table S1 Maternally DNA methylation-imprinted genes & maternally H3K27me-imprinted genes**
