# Supplementary figures and images for: Transcriptome Dynamics and Cell Dialogs Between Oocytes and Granulosa Cells in Mouse Follicle Development
Source: Genomics Proteomics Bioinformatics. 2023 Dec 6;22(2):qzad001. doi: 10.1093/gpbjnl/qzad001 (PMC11423849; doi:10.1093/gpbjnl/qzad001)

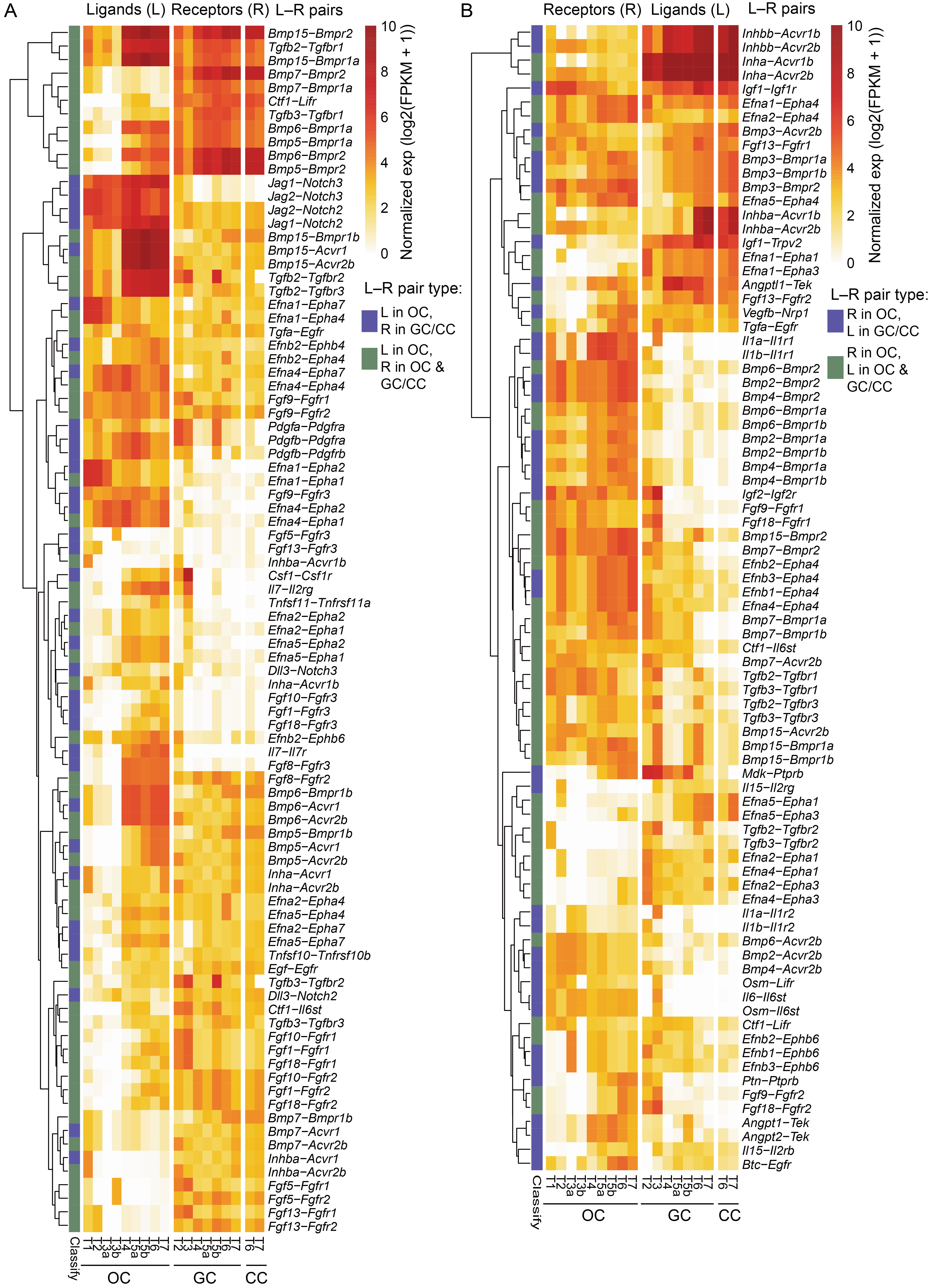

Supplement: qzad001_Supplementary_Data [file qzad001_supplementary_data.zip › FIGS5.jpg]

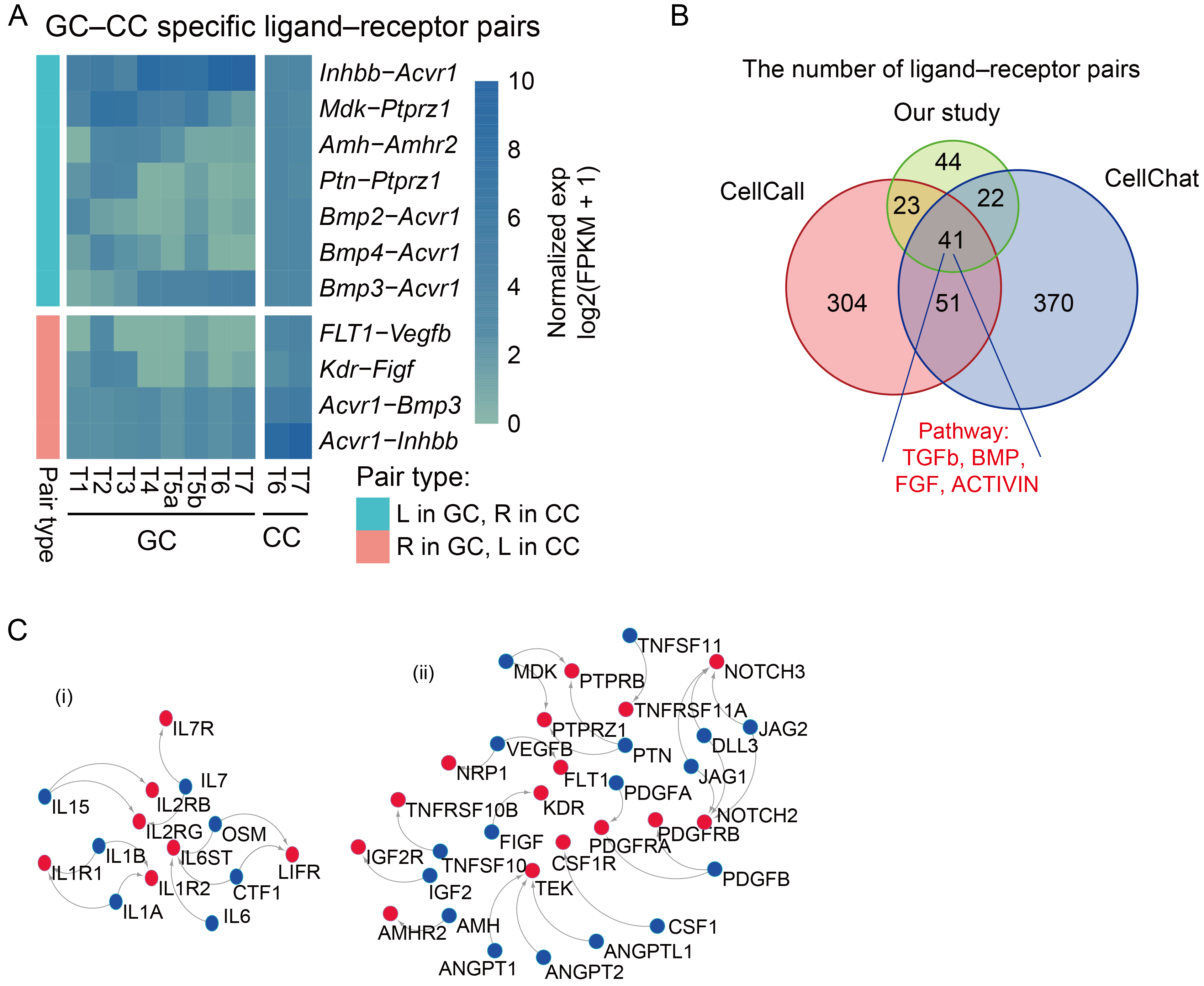

Supplement: qzad001_Supplementary_Data [file qzad001_supplementary_data.zip › FIGS6.jpg]

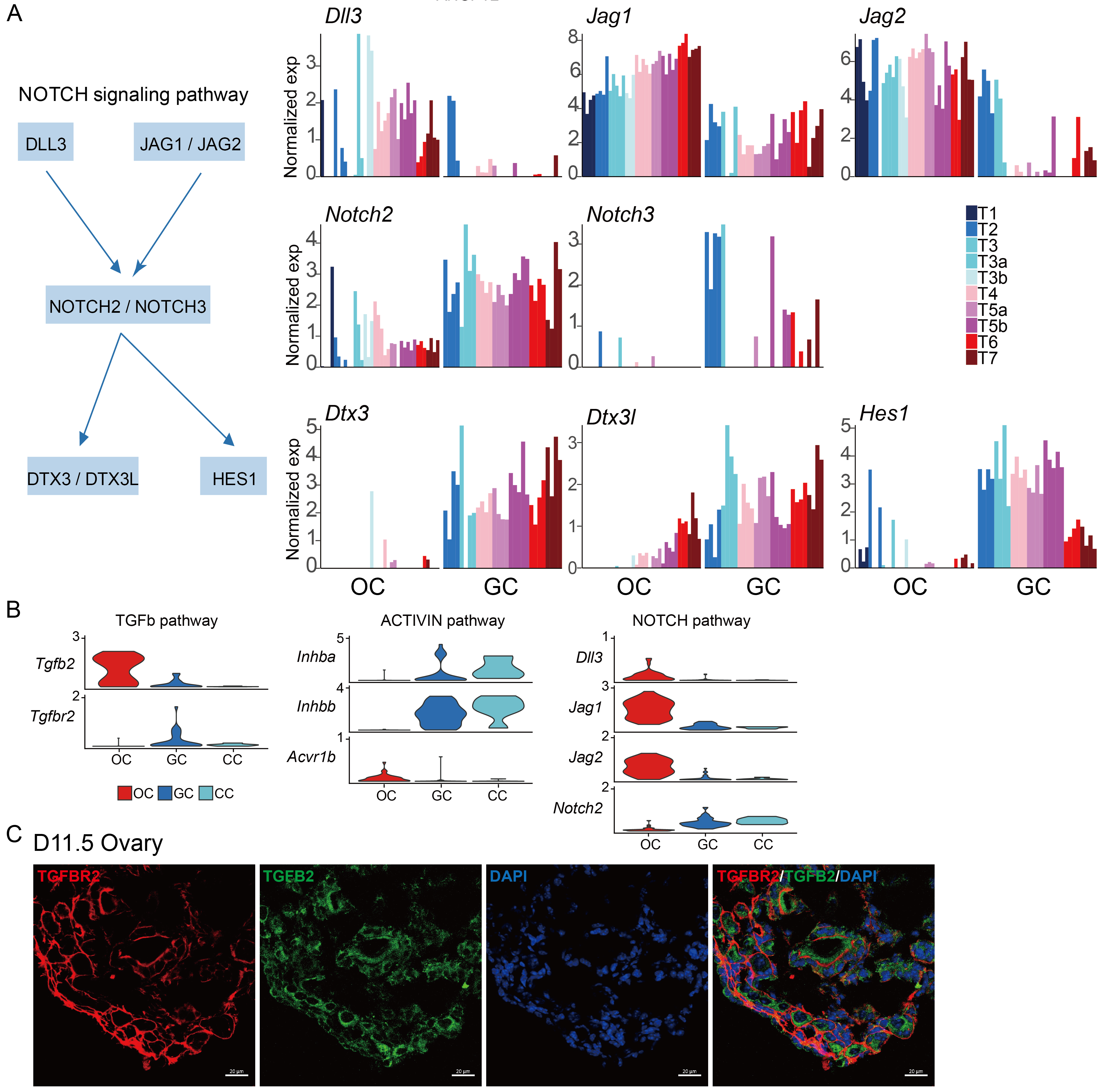

Supplement: qzad001_Supplementary_Data [file qzad001_supplementary_data.zip › FIGS7.jpg]

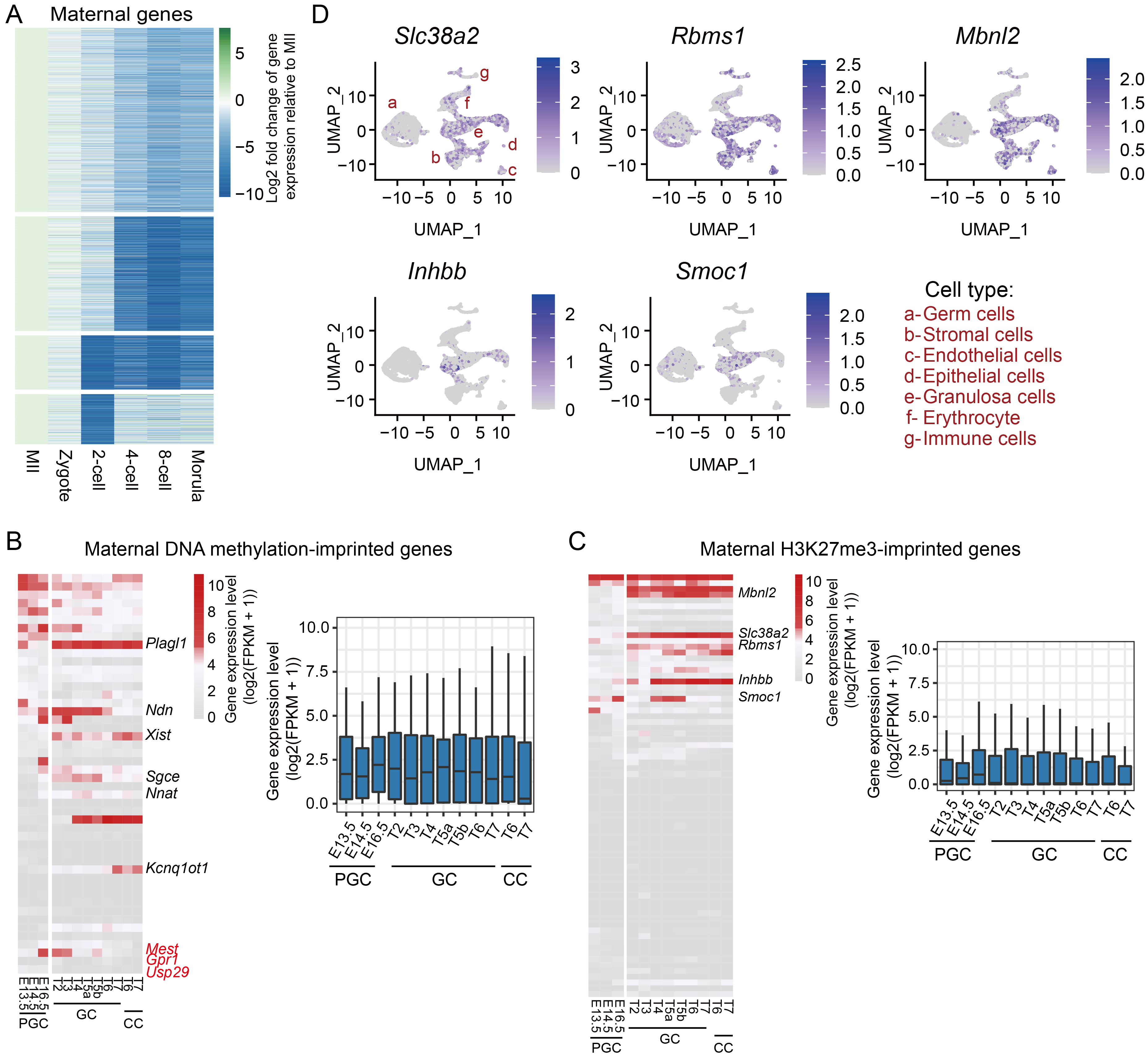

Supplement: qzad001_Supplementary_Data [file qzad001_supplementary_data.zip › FIGS8.jpg]

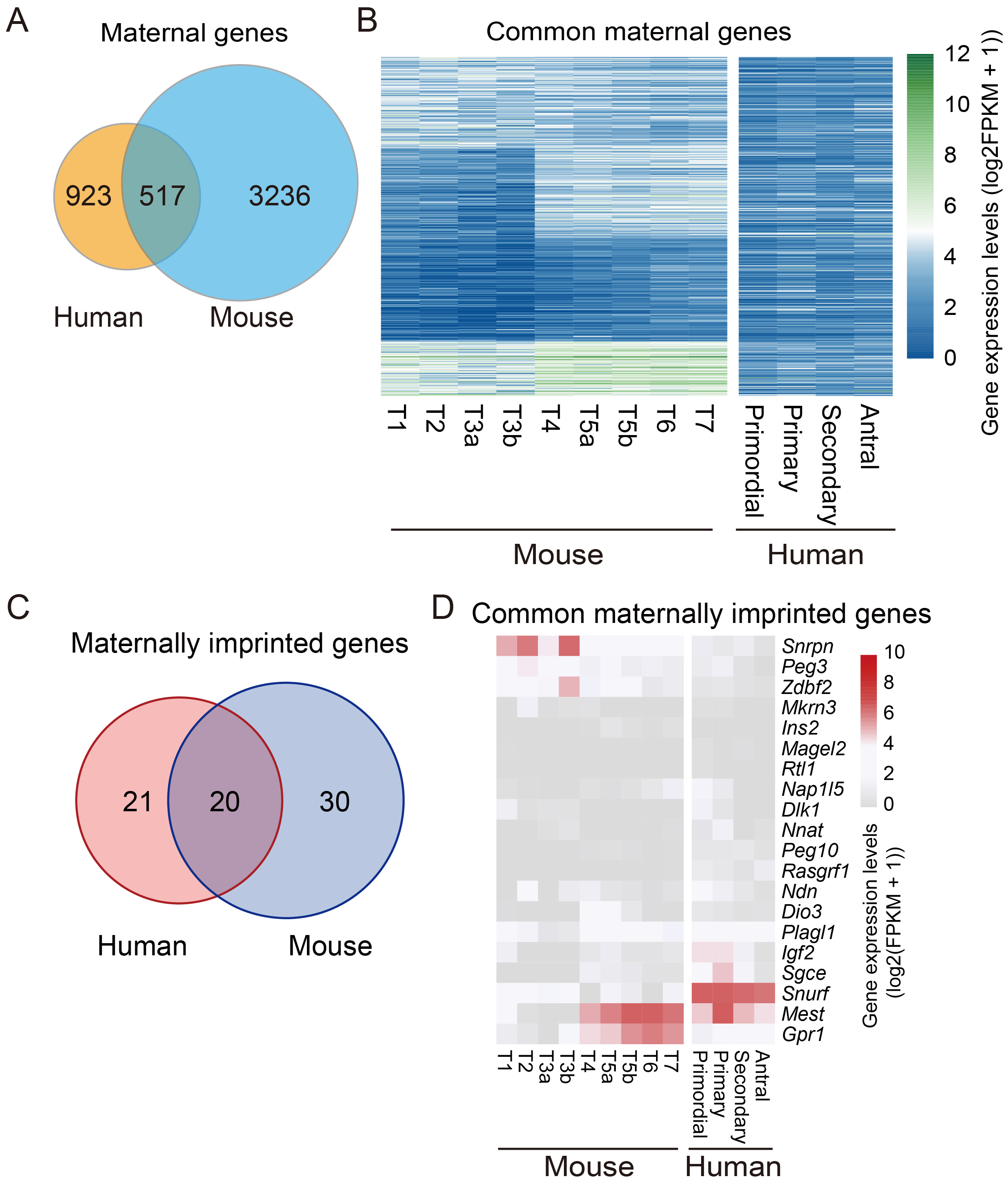

Supplement: qzad001_Supplementary_Data [file qzad001_supplementary_data.zip › FIGS9.jpg]

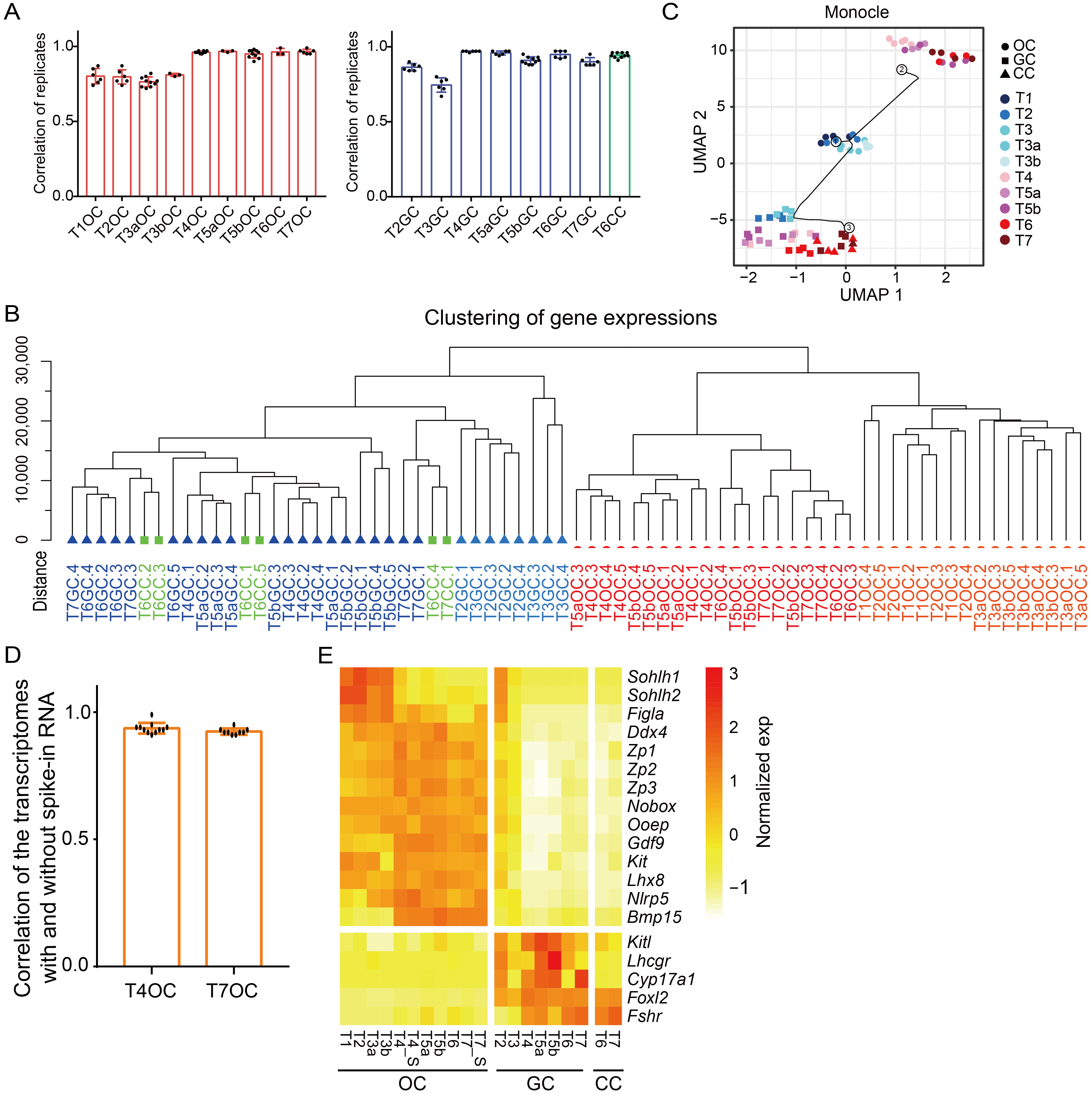

Supplement: qzad001_Supplementary_Data [file qzad001_supplementary_data.zip › FIGS1.jpg]

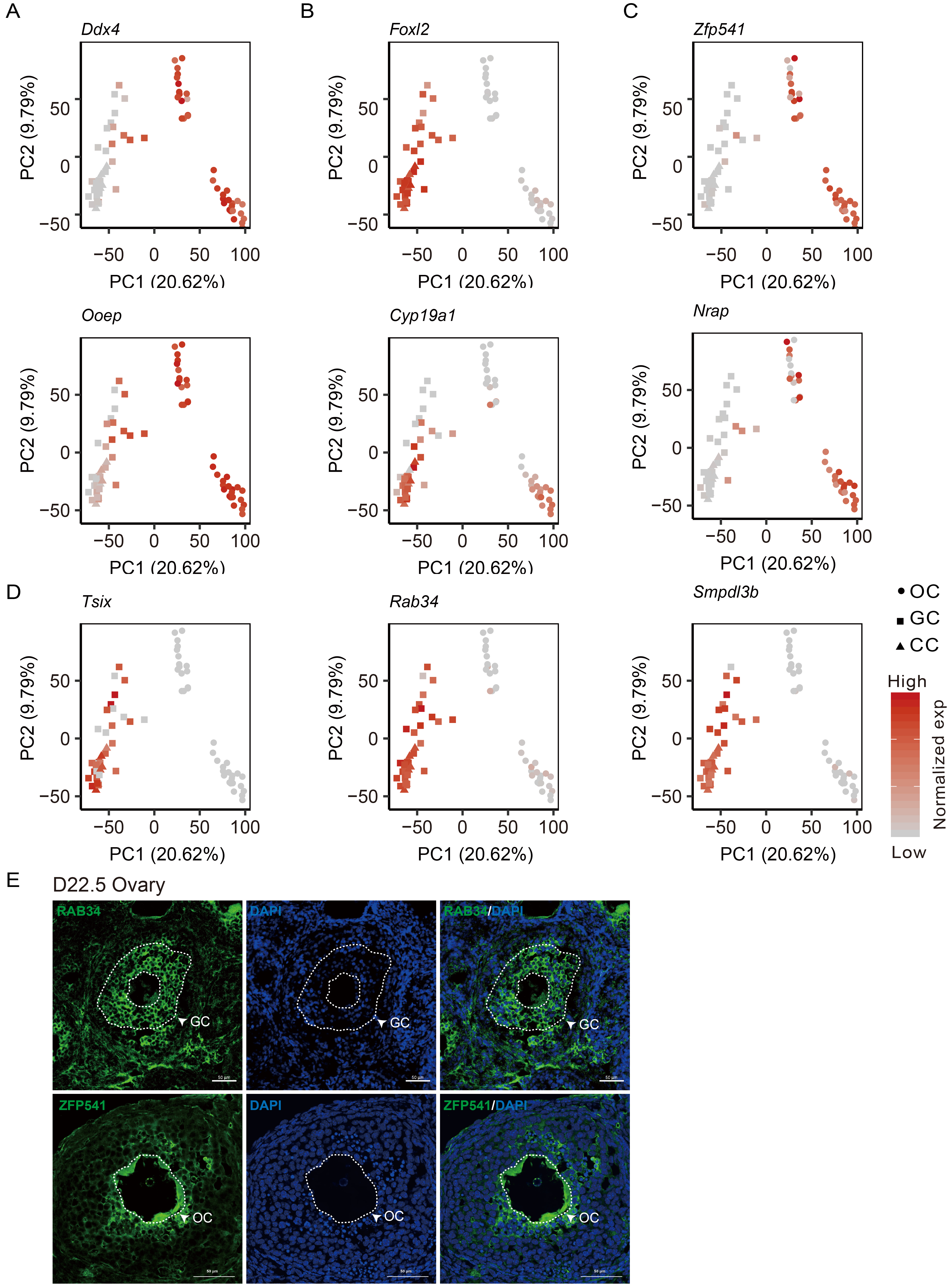

Supplement: qzad001_Supplementary_Data [file qzad001_supplementary_data.zip › FIGS2.jpg]

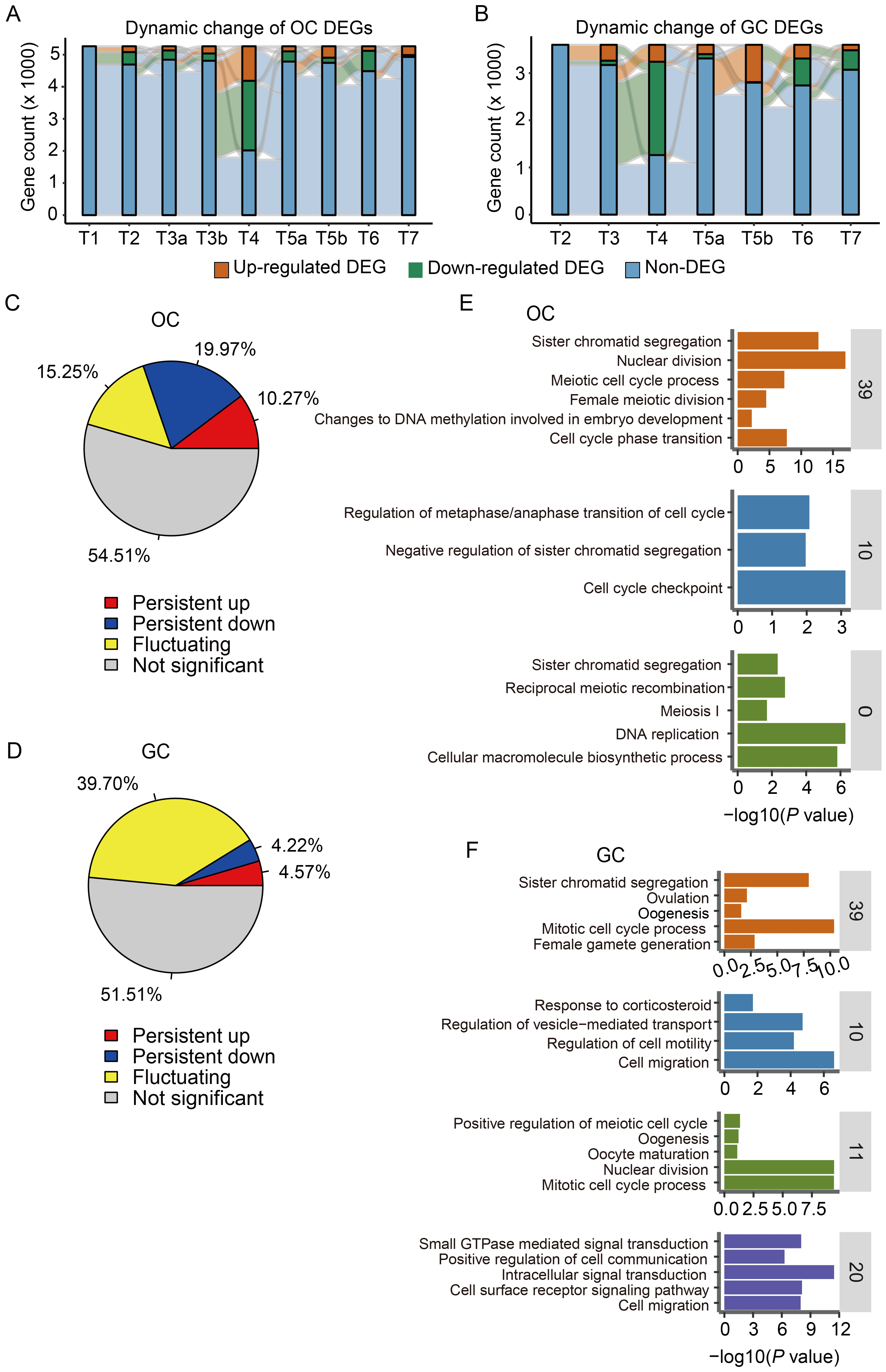

Supplement: qzad001_Supplementary_Data [file qzad001_supplementary_data.zip › FIGS3.jpg]

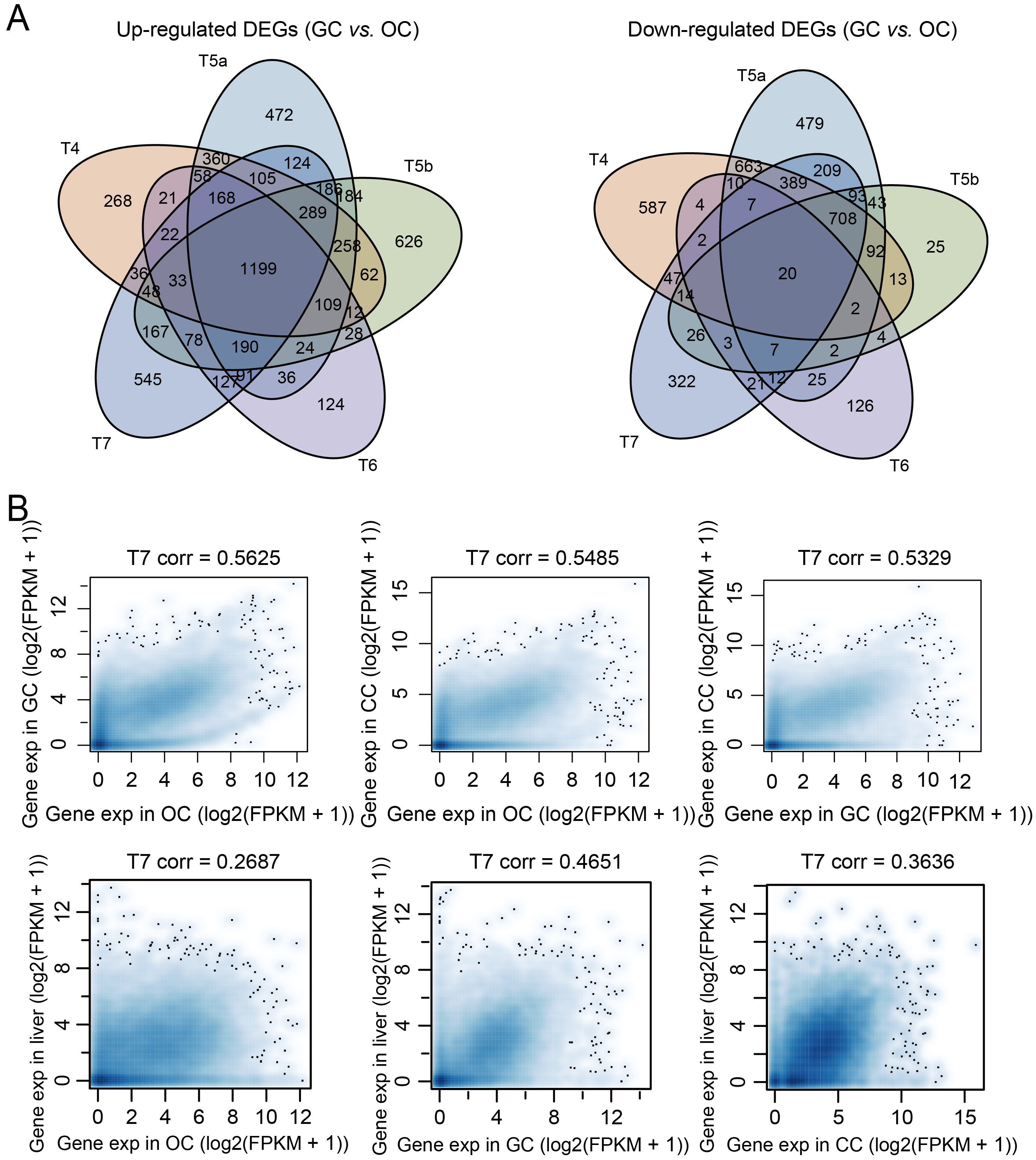

Supplement: qzad001_Supplementary_Data [file qzad001_supplementary_data.zip › FIGS4.jpg]
